# Household crowding hampers mitigating the transmission of SARS-CoV-2

**DOI:** 10.1590/0037-8682-0821-2020

**Published:** 2021-02-10

**Authors:** Daniel Antunes Maciel Villela

**Affiliations:** 1 Fundação Oswaldo Cruz, Programa de Computação Científica, Rio de Janeiro, RJ, Brasil.

**Keywords:** SARS-CoV-2, COVID-19, Epidemiology, Household crowding

## Abstract

**INTRODUCTION:**

Household crowding deserves attention when evaluating the transmission intensity of SARS-CoV-2 in Brazil. We aimed to evaluate the association between household crowding and COVID-19 incidence.

**METHODS:**

Linear and Poisson regression analyses were used to assess the associations between indices of household crowding (high, average, low) and COVID-19 incidence estimates.

**RESULTS:**

Cities with a high index of household crowding were linked with a significantly higher COVID-19 incidence estimate (excess of 461 per 100,000; 95% confidence interval: 371-558 per 100,000).

**CONCLUSIONS:**

Crowding typically promotes virus transmission. Considering urban and housing structures is essential in designing mitigation strategies during a pandemic.

Sustained transmission of severe acute respiratory syndrome coronavirus 2 (SARS-CoV-2) in Brazil started in large urban centers such as São Paulo and Rio de Janeiro; however, serological surveys that have been described and analyzed by Hallal et al*.* showed an early profound impact on cases and mortality rates in the North and Northeast regions^1^. Hallal et al*.* found heterogeneous prevalence estimates of SARS-CoV-2 antibodies across cities, including in the north of Brazil along the Amazon river, with 11 municipalities having antibody prevalence estimates greater than 2%^1^. The basic reproduction number (*R*_*0*_ ) estimated by Souza et al*.* was 2.6 [95% credible interval: 2.0-4.5] in the state of Amazonas^2^. Candido et al*.* evaluated the spread of SARS-CoV-2 in the country and found a reproduction number (*R*) *of >*3 in the early days of the pandemic in major centers before the adoption of non-pharmaceutical interventions (NPIs)^3^. These numbers indicated a high transmission intensity in the absence of actions for mitigation^3^ ^,^ ^4^, which later varied substantially owing to the states’ and cities’ interventions. However, the effects of other factors, such as household crowding, *i.e.* when the number of residents is usually large for a given physical space^5^, on these marked differences in the transmission intensity across the country require further investigation. Household crowding is an important factor to consider given the living conditions in Brazil. In particular, it is necessary to analyze whether such factors hamper attempts to mitigate SARS-CoV-2 transmission.

The household crowding hypothesis was analyzed using the number of coronavirus disease (COVID-19) cases in municipalities of Brazil and Brazilian census data on population and housing conditions. Census data (2010), which are maintained by the National Institute of Geography and Statistics (IBGE), provided the population sizes of cities and number of households using the following categories: 1 resident per room; 1-2 residents per room; 2-3 residents per room; and > 3 residents per room. For each municipality, the proportion of households with > 3 residents per room that is used as a bedroom was the index utilized to classify Brazilian municipalities by levels of household crowding. 

The distribution of the proportion of households with ≥ 3 residents per room that is used as a bedroom provided the thresholds for municipality classifications into low, average, and high crowding levels. The proportion of households with ≥ 3 residents per room from the census data revealed a lognormal distribution, with a mean of 3.35%. Household crowding was classified into the following levels: low (<2.17% [difference between the mean proportion and half the standard deviation]), high (>5.18% [sum of the mean proportion and half the standard deviation]), and average (>2.17% and <5.18%). Therefore, 28.60% (1,583) of the municipalities were classified as low, 31.60% (1,749) as high, and 39.70% (2,200) as average.

The cumulative incidence of COVID-19 in the municipalities of Brazil was calculated as the ratio between the number of confirmed cases up to June 10, 2020 per municipality^6^ and city population (IBGE). The mean value of the cumulative incidence in the given period was 88 cases per 100,000 inhabitants. Figure 1 shows that municipalities with a high index of household crowding had higher incidence estimates, whereas those with low and average indices had very similar distributions of cumulative incidence estimates. A linear regression analysis that used the incidence estimate as the outcome and household crowding index (low, average, high) as the explanatory variable revealed that the group with a high index of household crowding was associated with a significantly higher COVID-19 incidence (an excess of 461 persons per 100,000; 95% confidence interval (CI): 371-558 cases per 100,000; p-value<0.001) than the group with a low index of household crowding. Most high-index municipalities with confirmed cases were in the northern region (150 cities) and northeast region (198 cities), followed by regions in the Southeast (53 cities), Center-West (6 cities), and South (6 cities). In addition, a Poisson regression analysis that used the cumulative number of confirmed cases as the outcome, proportion of households with ≥ 3 residents per room as the explanatory variable (log-transformed), and city population as the offset (log-transformed) revealed a significantly increasing effect of household crowding on COVID-19 incidence (factor: 0.824, 95% CI: 0.819-0.831). 

**FIGURE 1:**
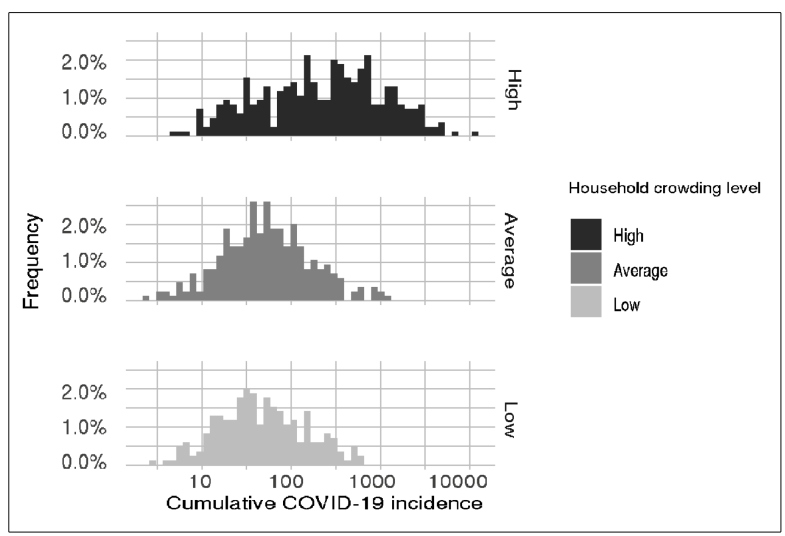
Distribution of cumulative COVID-19 incidence across municipalities according to the different levels of household crowding (low, average, and high).

These findings showed how household crowding may adversely affect the incidence of COVID-19. In the early weeks of the epidemic in Brazil, only NPIs were available to reduce virus transmission; however, household crowding likely hampered the effectiveness of such measures. The role of household crowding, an important topic listed by the World Health Organization Housing and Health Guidelines, has been previously recognized in the transmission of infectious diseases^5^ ^,^ ^7^. However, the effectiveness of mitigating actions varies across cities and states. Consequently, the impact on mitigating transmission (“flattening the curve”) also differed across the country; the implementation of interventions could have been limited in a few cities, including in those cities with high household crowding. Moreover, the distribution of household crowding in Brazil might have changed since the most recent census in Brazil in 2010. However, a substantial part of the category of cities with high household crowding may still be significantly above the mean proportion of crowding. Analysis of antibody prevalence by Hallal et al*.* in surveys conducted 3 weeks apart indicated higher antibody prevalence estimates in households having > 6 persons. 

In summary, many factors play essential roles in increasing the transmission intensities of SARS-CoV-2 worldwide, including in Brazil. Hallal et al*.* found considerable variability in two early serological surveys, pointing to various heterogenous outcomes according to ethnicity, income levels, and household size. Therefore, there are potential factors that may be considered as confounding variables and warrant further research, namely sociodemographic structures in municipalities, as well as local adherence to mitigation strategies. Grassly et al*.* found a significant reduction in reproduction numbers after molecular testing for screening and contact tracing was used; this potentially might have reduced transmission in crowded households^8^. Furthermore, modeling studies have assessed the impact of high transmission within households as the secondary attack rate^9^ ^,^ ^10^. The findings in this study demonstrated that household crowding may be a potential factor that could hamper transmission mitigation measures by evaluating the link between cumulative COVID-19 incidence and an indicator of household crowding. Therefore, this factor may be essential to consider while defining surveillance strategies such as contact tracing.
